# Chemotactic Host-Finding Strategies of Plant Endoparasites and Endophytes

**DOI:** 10.3389/fpls.2020.01167

**Published:** 2020-07-31

**Authors:** Allen Yi-Lun Tsai, Morihiro Oota, Shinichiro Sawa

**Affiliations:** Graduate School of Science and Technology, Kumamoto University, Kumamoto, Japan

**Keywords:** chemotaxis, endophytes, endoparasites, gall-forming bacteria, arbuscular mycorrhizal fungi, plant pathogenic nematode

## Abstract

Plants interact with microorganisms in the environment during all stages of their development and in most of their organs. These interactions can be either beneficial or detrimental for the plant and may be transient or long-term. In extreme cases, microorganisms become endoparastic or endophytic and permanently reside within a plant, while the host plant undergoes developmental reprogramming and produces new tissues or organs as a response to the invasion. Events at the cellular and molecular level following infection have been extensively described, however the mechanisms of how these microorganisms locate their plant hosts *via* chemotaxis remain largely unknown. In this review, we summarize recent findings concerning the signalling molecules that regulate chemotaxis of endoparasitic/endophytic bacteria, fungi, and nematodes. In particular, we will focus on the molecules secreted by plants that are most likely to act as guidance cues for microorganisms. These compounds are found in a wide range of plant species and show a variety of secondary effects. Interestingly, these compounds show different attraction potencies depending on the species of the invading organism, suggesting that cues perceived in the soil may be more complex than anticipated. However, what the cognate receptors are for these attractants, as well as the mechanism of how these attractants influence these organisms, remain important outstanding questions. Host-targeting marks the first step of plant—microorganism interactions, therefore understanding the signalling molecules involved in this step plays a key role in understanding these interactions as a whole.

## Introduction

Plants do not live in solitary isolation but instead are constantly interacting with other organisms in their environment. Organisms known to interact with plants include herbivores, commensals, symbionts, and pathogens from multiple kingdoms. These organisms can interact with essentially any plant organ throughout all stages of plant development. Certain plant parasites and symbionts infect host-plant tissues and spend the majority of their lives within their host (Compant et al., 2010; Hassani et al., 2018). Such manipulation of host development has evolved independently several times and can be found in multiple classes of organisms, including bacteria, fungi, nematodes, mites, and insects (Barash and Manulis-Sasson, 2009; Dodueva et al., 2020).

Plant endoparasite/endophyte-induced structures can have profound effects in agriculture. Colonization by symbionts usually grants certain advantages to the host plant, such as enhanced nutrient acquisition, and is thus generally preferred (if not required) in agriculture (Khare et al., 2018). On the other hand, parasite-induced ectopic structures are typically signs of disease that reduce crop performance, and can sometimes be fatal. However, what remains unclear is how these organisms locate their hosts. Despite plants being sessile, endoparasites and endophytes nevertheless need to make an effort to locate their host plants. Some endoparasites and endophytes have very specific host ranges, while for others plant hosts are obligatory to complete their life cycles. As such, host-seeking is clearly a vital behavior in plant endoparasites and endophytes and one that requires intricate regulation. It is generally accepted that to locate host plants, endosymbionts and endoparasites sense attractants secreted by these plants. However, the mechanisms by which these attractants are perceived and identified generally remain unclear.

This review aims to explore the current status the chemotactic behaviors of plant endoparasite/endophyte, particularly those that induce host-plant structural remodeling. The chemotactic behavior and chemosensory mechanisms of bacteria, fungi, and nematodes toward plants will be introduced, summarizing chemotactic signaling systems established in these respective taxa using model organisms. The chemotactic mechanisms and known attractants for plant-infecting members of each taxon will then be discussed.

## Endoparasitic and Endophytic Bacteria

The best-characterized examples of plant-infecting organism-induced plant developmental remodeling are caused by bacteria. *Rhizobium radiobacter*, the causative agent of crown gall disease, stimulates tumor formation on the shoots and roots of many plant species, while various rhizobia species colonize plant roots and form nodules to provide organic nitrogen in exchange for carbohydrates (Escobar and Dandekar, 2003; Poole et al., 2018). Multiple species of bacteria have been shown to migrate toward root exudates, and the rhizosphere is indeed known to be colonized by many species of microorganisms (Walker et al., 2003; Berendsen et al., 2012). However, the specific components within root exudates that soil bacteria respond to largely remain to be deciphered. In addition, exudate compositions also vary among root regions, adding temporal and spatial variations to bacterial behavior in the rhizosphere (Scharf et al., 2016). Lastly, root exudates can mediate bacterial colonization not only through chemotaxis but also through other means, such as promoting nodulation or inducing flagellin expression (Kierul et al., 2015; Li et al., 2016).

Chemotaxis has been well-characterized in the model organism *Escherichia coli*. The perception of chemotactic signals in *E. coli* is mediated by the core complex, which consists of four methyl-accepting chemotaxis proteins (MCPs) that act as chemoreceptors, and redox receptor Aer, histidine kinase CheA and adaptor protein CheW (Yang and Briegel, 2020). The core complexes in turn form large hexagonal clusters on the plasma membrane, known as the chemosensory array, and are responsible for phosphorylating downstream signalling modules upon chemoattractant binding (Yang and Briegel, 2020). Downstream targets of the core complex include CheB, which mediates sensory adaptation and inhibits the MCPs as a negative feedback signal, and CheY, which controls flagella-mediated locomotion (Wadhams and Armitage, 2004). By favoring long-flagella-mediated propulsion in the presence of chemoattractants, the bacterial cell gradually moves closer to the attractant.

The number of chemoreceptors and the tertiary structures of the core complex show great diversity among bacterial taxa, although in general the chemotactic machinery seen in *E. coli* is well-conserved among bacteria and serves as a suitable model system (**Table 1**). Currently, 19 bacterial chemotaxis systems have been identified; 17 based on the *E. coli* Che system, with two other unique systems known as type IV pili motility (Tfp) and alternative cellular function (ACF) (Wuichet and Zhulin, 2010). More than half of the motile bacteria possess multiple chemosensory systems, highlighting the importance of processing and fine-tuning chemosensory signalling and responses (Wuichet and Zhulin, 2010). Expectedly, the number and diversity of MCPs expressed in a given taxon correlate with its lifestyle and metabolism complexity (Lacal et al., 2010). Several species of soil bacteria have been documented to be attracted by organic acids, for which the cognate chemoreceptors have been identified in many species (Sampedro et al., 2015; **Table 2**). Other common bacterial chemoattractants include sugars and sugar alcohols (Bowra and Dilworth, 1981; Burg et al., 1982; Alexandre et al., 2000; Meier et al., 2007; Miller et al., 2007; **Table 2**).

**Table 1:** Chemotactic genes of endoparasites and endophytes discussed in this review.

Endoparasite, endophyte	Chemotactic gene	Model organism orthologues	Predicted functions	Reference
*Rhizobium radiobacter* (bacteria)	ChvE	*E. coli* galactose/glucose-binding protein (GBP)	Putative sugar chemoreceptor	Cangelosi et al., 1990
	CheW_1_, CheW_2_	*E. coli* CheW	Scaffold protein binding chemoreceptor and histidine kinase CheA	Huang et al., 2018
*Rhizobium leguminosarum* (bacteria)	McpB, McpC	*E. coli* MCPs	Chemoreceptors with unknown ligands	Yost et al., 1998
*Sinorhizobium meliloti* (bacteria)	McpE, McpS, McpT, McpU, McpV, McpW, McpX, McpY, McpZ	*E. coli* MCPs	Chemoreceptors for sugars, amino acids and organic acids	Meier et al., 2007
	CheY1, CheY2	*E. coli* CheY	Binds and changes the rotation direction of flagellar motor,	Sourjik and Schmitt, 1996; Sourjik and Schmitt, 1998
	CheD	*E. coli* CheD	Deaminase that regulates chemoreceptor activities	Scharf et al., 2016
	CheS	N/A	Regulates phosphorylation of CheY1	Dogra et al., 2012
	CheT	N/A	Required for chemotaxis, function unknown	Scharf et al., 2016
*Fusarium oxysporum* (fungi)	STE2	*S. cerevisiae* Ste2	Chemoreceptor for unknown host signal	Turrà et al., 2015
	Fmk1	*S. cerevisiae* Fus3 and Kss1	MAP kinase for chemotropism signaling,	Di Pietro et al., 2001
*Meloidogyne incognita* (nematode)	Mi-odr-1	*C. elegans* odr-1	Membrane-bound guanylyl cyclase that produces cGMP secondary messenger	Shivakumara et al., 2019
	Mi-odr-3	*C. elegans* odr-3	Gα protein that regulates cyclic nucleotide metabolism	Shivakumara et al., 2019
	Mi-tax-2, Mi-tax-4	*C. elegans* tax-2 and tax-4	Subunits of cyclic nucleotide-gated cation channel involved in G-protein-mediated signalling	Shivakumara et al., 2019

**Table 2:** Chemoattractants of endoparasites and endophytes discussed in this review.

Attractant class	Perceived by	Attractants	Notes	References
Sugars and alcohols	*Rhizobium radiobacter* (bacteria)	Sucrose, glucose, fructose, maltose, lactulose, galactose, raffinose, stachyose, arabinose	May be perceived by chemoreceptor ChvE	Loake et al., 1988; Cangelosi et al., 1990
	*Rhizobium leguminosarum* (bacteria)	Mannitol, galactose	Perception requires the Che1 chemotaxis operon	Miller et al., 2007
	*Sinorhizobium meliloti* (bacteria)	Fructose, galactose, maltose, mannitol, sucrose	Perception requires all 9 chemoreceptors McpE, McpS-McpZ	Meier et al., 2007
		Sucrose, glucose, arabinose, galactose		Malek, 1989
	*Meloidogyne incognita* (nematode)	Mannitol	Signal transduction may require *Mi-odr-1*, *Mi-odr-3*, *Mi-tax-2* and *Mi-tax-4*	Fleming et al., 2017; Shivakumara et al., 2019
Organic acids	*Rhizobium leguminosarum* (bacteria)	Pyruvate, succinate	Perception requires the Che1 chemotaxis operon	Miller et al., 2007
	*Sinorhizobium meliloti* (bacteria)	Citrate, fumarate, malate, succinate	Perception requires all 9 chemoreceptors McpE, McpS-McpZ	Meier et al., 2007
	*Meloidogyne incognita* (nematode)	Vanillic acid, lauric acid	Signal transduction may require *Mi-odr-1*, *Mi-odr-3*, *Mi-tax-2* and *Mi-tax-4*.	Dong et al., 2014; Fleming et al., 2017; Shivakumara et al., 2019
Amino acids	*Rhizobium radiobacter* (bacteria)	Valine, arginine		Loake et al., 1988
	*Rhizobium leguminosarum* (bacteria)	Homoserine		Armitage et al., 1988
	*Sinorhizobium meliloti* (bacteria)	All standard amino acids	Perception requires all 9 chemoreceptors McpE, McpS-McpZ	Götz et al., 1982; Malek, 1989; Meier et al., 2007; Webb et al., 2014; Webb et al., 2017a
		Citrulline, γ-aminobutyric acid, ornithine	Perception requires chemoreceptor McpU	Webb et al., 2017a
		Homoserine		Götz et al., 1982
	*Meloidogyne incognita* (nematode)	Argenine, lysine	Signal transduction may require *Mi-odr-1*, *Mi-odr-3*, *Mi-tax-2* and *Mi-tax-4*	Fleming et al., 2017; Shivakumara et al., 2019
Phenolics	*Rhizobium radiobacter* (bacteria)	Acetosyringone, sinapinic acid, syringic acid		Ashby et al., 1987; Ashby et al., 1988
	*Meloidogyne incognita* (nematode)	Tannic acid	Signal transduction may require *Mi-odr-1*, *Mi-odr-3*, *Mi-tax-2* and *Mi-tax-4*	Fleming et al., 2017; Shivakumara et al., 2019
Flavonoids	*Rhizobium leguminosarum* (bacteria)	Apigenin, naringenin, kaempferol		Armitage et al., 1988
	*Sinorhizobium meliloti* (bacteria)	Luteolin, 4’,7-dihydroxyflavone, 4’,7-dihydroxyflavanone, and 4,4’-dihydroxy-2-methoxychalcone		Caetano-Anollés et al., 1988; Dharmatilake and Bauer, 1992
Phyto-hormones	*Gigaspora margarita* (fungi)	Strigolactone	Likely perceived by novel receptors not conserved in plants.	Akiyama et al., 2005; Akiyama and Hayashi, 2006; Gutjahr, 2014; Boyer et al., 2014
	*Meloidogyne incognita* (nematode)	6-Dimethylallylaminopurine, salicylic acid, gibberellic acid, Indole-3-acetic acid	Signal transduction may require *Mi-odr-1*, *Mi-odr-3*, *Mi-tax-2* and *Mi-tax-4*	Fleming et al., 2017; Shivakumara et al., 2019
Organic amines	*Sinorhizobium meliloti* (bacteria)	Betonicine, choline, glycine betaine, stachydrine, trigonelline	Perception requires chemoreceptor McpX	Webb et al., 2017b
	*Meloidogyne incognita* (nematode)	Cadaverine, 1,3-diaminopropane, putrescine		Oota et al., 2019
Opines	*Rhizobium radiobacter* (bacteria)	Octopine, nopaline, mannopine, agrocinopines A+B		Kim and Farrand, 1998
Others	*Rhizobium leguminosarum* (bacteria)	Unknown host signal	Perception requires chemoreceptors McpB and McpC	Yost et al., 1998
	*Fusarium oxysporum* (fungi)	Unknown host signal	Perception requires α-STE2 chemoreceptor and Fmk1 MAPK kinase, signal requires peroxidase activity from host	Turrà et al., 2015
	*Trichoderma harzianum* (fungi)	Unknown host signal	requires stress, peroxidase and oxylipin activities from host	Lombardi et al., 2018
	*Meloidogyne incognita* (nematode)	Calcium chloride		Wang et al., 2018

*Rhizobium radiobacter* (formerly known as *Agrobactetrium tumefaciens*), the causative agent of crown gall disease, is perhaps the best-known endoparasitic organism that manipulates plant development. *R. radiobacter* probably targets molecules specifically released by wounding, since it infiltrates plant tissues *via* wound sites. As such, *R. radiobacter* has been shown to be attracted to various sugars, amino acids, opines, and phenolics (Ashby et al., 1987; Ashby et al., 1988; Loake et al., 1988; Kim and Farrand, 1998). One of the chemoreceptors, ChvE, has been shown to be essential for host-finding and shares structural homology with *E. coli* proteins known to bind galactose and glucose, suggesting ChvE may similarly function as a chemoreceptor for sugars (Cangelosi et al., 1990). Interestingly, *R. radiobacter* expresses two CheW homologues, both of which are required for chemotaxis towards plant tissue, yet neither is encoded in the Che operon (Huang et al., 2018).

*Rhizobium leguminosarum* is one of the best characterized rhizobia and is related to *R. radiobacter*; they both belong to the Rhizobiaceae family. *R. leguminosarum* forms nodules in the roots of legumes, such as peas, clovers, and various beans, and is an important contributor to nitrogen fixation. Its genome contains two chemotaxis operons, where Che1 is likely to be the main driver mediating chemotaxis toward sugars and is essential for host-finding and nodulation (Miller et al., 2007). *R. leguminosarum* has been shown to be attracted to amino acids and flavonoids (Armitage et al., 1988). In addition, two of its chemoreceptors, McpB and McpC, are known to positively regulate nodulation, but their ligands remain unknown (Yost et al., 1998). The importance of these two receptors may be more relevant depending on the host species and competing soil microbiota (Yost et al., 1998).

*Sinorhizobium meliloti* is another well-characterized member of the Rhizobiaceae family. *S. meliloti* has been shown to colonize specific regions of alfalfa roots, confirming their preference for cues from specific parts of the roots (Gulash et al., 1984). The *S. meliloti* genome contains nine chemoreceptors, all of which were shown to be required for chemotaxis toward sugars, amino acids, and organic acids (Meier et al., 2007). Two CheY homologues are also present, with CheY2 controlling the unidirectional flagella motor speed, while CheY1 terminates the chemotaxis signal (Sourjik and Schmitt, 1996; Platzer et al., 1997; Sourjik and Schmitt, 1998; Attmannspacher et al., 2005). The chemotactic machineries are encoded in two operons (Meier et al., 2007; Meier and Scharf, 2009). The Che1 operon of *S. meliloti* contains the CheD deamidase that modulates chemoreceptor activities (Scharf et al., 2016). *S. meliloti* also expresses CheS, a novel protein that complexes with CheA to facilitate dephosphorylation of CheY1 (Dogra et al., 2012). CheT is another novel protein in *S. meliloti* Che1 operon required for chemotaxis, though its function is currently unknown (Scharf et al., 2016). *S. meliloti* has been documented to be attracted to luteolin, 4’,7-dihydroxyflavone, 4’,7-dihydroxyflavanone, and 4,4’-dihydroxy-2-methoxychalcone, all of which are found in root exudates (Caetano-Anollés et al., 1988; Dharmatilake and Bauer, 1992). In addition, *S. meliloti* has been shown to be attracted to amino acids in alfalfa seed exudates, which is mediated by McpU, as well as to common sugars (Götz et al., 1982; Malek, 1989; Meier et al., 2007; Webb et al., 2014; Webb et al., 2017a). Other known *S. meliloti* attractants include quaternary ammonium compounds (betonicine, choline, glycine betaine, stachydrine, and trigonelline), which are recognized by McpX (Webb et al., 2017b).

## Endoparasitic and Endophytic Fungi

The other prominent class of organisms known to invade plant tissues is the fungi. Unlike bacteria, fungi are immobile and under most circumstances are not chemotactic. Nevertheless, plant-symbiotic and parasitic fungi make deliberate efforts to mediate hyphae growth toward potential hosts *via* chemotropism. Hyphae chemotropism towards plants was first described in *Uromyces appendiculatus* growing towards soybean leaf stomata, with the tips of hyphae recognized as the area responsible for sensing chemical cues and processing chemotropism (Turrà and Di Pietro, 2015; Turrà et al., 2016). By 1905, it was noted that the constituents of host exudates dictated the type of fungi attracted, which consolidates the importance of chemotaxis in plant parasitism (Massee, 1905).

In a similar way to how *E. coli* serves as a model for bacterial chemotaxis, studies using *Saccharomyces cerevisiae* and *Neurospora crassa* have provided invaluable insights into fungal chemotropism (**Table 1**). *S. cerevisiae* cells develop mating projections known as shmoos in the presence of the opposite mating type, by detecting secreted mating peptide pheromone a or α. These pheromones are perceived by seven transmembrane G-protein-coupled receptors; MATa cells express Ste2, which binds the α- pheromone, while MATα cells express Ste3, which binds the a-pheromone (Hagen et al., 1986; Blumer et al., 1988). The receptors function as guanine exchange factors and activates the Gα subunit (GPA1) upon pheromone-binding, which promotes the dissociation of the Gβγ subunits (STE4 and STE18) from the complex (Schrick et al., 1997). This then initiates a signalling cascade mediated by Fus3 and Kss1 (MAPK), leading to transcriptional regulation, cell cycle arrest, cell shape alternation, and ultimately shmoo development toward the mating partner (Arkowitz, 2009). In an analogous case, female hyphae of *Neurospora crassa* (trichogyne) grow towards male spores *via* chemotropism. This process is mediated by the spore pheromone peptides MFA-1 and CCG-4, which are perceived by the receptors PRE-1 and PRE-2 (orthologues of Ste2 and Ste3), respectively (Kim and Borkovich, 2004; Kim and Borkovich, 2006). Pheromone perception in *N. crassa* initiates a similar MAPK signalling cascade mediated by heterotrimeric G-proteins (Dettmann et al., 2014). Another case of chemotropism in *N. crassa* is anastomosis, where hyphae from cells of an identical genotype (sometimes the same cell) are attracted towards each other, followed by fusion (Leeder et al., 2011). The anastomosis chemotropism signal is similarly transduced by a MAPK cascade using orthologues of Fus3 and Kss1 (Read et al., 2009). The *N. crassa* anastomosis signal may be a peptide pheromone (Roca et al., 2005), and it has been hypothesized that both parties use the same signalling molecule, which positively regulates itself (Read et al., 2012). Lastly, hyphae repellants may also play a role in chemotropism, and the direction of hyphae growth is likely to be a balance between attraction and repulsion (Leeder et al., 2011).

Hyphal chemotropism in response to plants is well-characterized in pathogens of the genus *Fusarium*, which are ubiquitous, filamentous ascomycete fungi. *Fusarium oxysporum* spores respond to host cues in order to germinate, and its hyphae elongate toward host roots using chemotropism. Although *F. oxysporum* does not manipulate the host’s developmental program, it nevertheless serves as a good model to decipher how yeast chemotropism has been specialized for pathogenesis. *F. oxysporum* requires the α-STE2 signalling module and Fmk1 (an orthologue of Fus3 and Kss1) for infection (Di Pietro et al., 2001; Turrà et al., 2015). Considering *F. oxysporum* does not undergo sexual reproduction, the conserved mating pheromone chemotropism pathway may have evolved to detect host signals (Turrà et al., 2015). *F. oxysporum* has been shown to be able to distinguish between live and dead cells, suggesting it is likely to be able to perceive certain live cell-exclusive signals (van der Does et al., 2008). Furthermore, *F. oxysporum* root-targeting behavior has been shown to require the secretion of a haem-containing peroxidase released from root wounds (Turrà et al., 2015), suggesting the product of this peroxidase may be a potential chemoattractant, in addition to nutrients such as amino acids and sugars. On the other hand, the biocontrol agent *Trichoderma harzianum* has been shown to be preferentially attracted to root exudate secreted by tomato plants under stress; peroxidase and oxylipins are required in the exudate for this attraction to occur (Lombardi et al., 2018). Interestingly, stress did not enhance the attraction of tomato root exudate to *F. oxysporum*, even though peroxidase has been shown to be an important element in *F. oxysporum* chemotropism (Turrà et al., 2015; Lombardi et al., 2018). The specific identities of the peroxidase-dependent attractants for *F. oxysporum* may be more complicated than expected.

The fungal counterparts of the bacterial rhizobia are the arbuscular mycorrhizal fungi (AMF). AMF include the Glomeromycetes, obligate symbionts that form highly branched structures known as arbuscules to mediate nutrient exchange with their host root’s cortical cells. AMF provide their plant hosts with various nutrients, predominantly inorganic phosphate, while receiving photosynthetic products such as hexoses and fatty acids from their host (Jiang et al., 2017; Luginbuehl et al., 2017). AMF have been estimated to colonize ~80% of all land plants, while fossil records suggest plant—AMF symbioses occurred as early as 460 million years ago, coinciding with the colonization of land by plants (Martin et al., 2017; Strullu-Derrien et al., 2018). These lines of evidence suggest AMF may be a key factor in plant terrestrial adaptation. AMF probably locate their host plants by recognizing molecules from root exudates, as root exudates have been shown to promote AMF spore germination and hyphal branching. The phytohormone strigolactone (SL) has been shown to promote hyphal branching in *Gigaspora margarita* (Akiyama et al., 2005; Akiyama and Hayashi, 2006; **Table 2**), while pea plants deficient in SL synthesis show reduced AMF colonization (Gómez-Roldán et al., 2008). Specifically, SL treatment stimulates AMF mitochondria proliferation and shape change, and increases metabolism (Besserer et al., 2006; Besserer et al., 2008; Besserer et al., 2009). SL also induces spore germination in AMF (Besserer et al., 2006; Besserer et al., 2008). Together, these lines of evidence confirm that secreted SL is indeed a vital positive regulator of AMF colonization. No fungal receptor of SL has yet been identified, but it is likely to be different from the plant SL receptor, since *G. margarita* perceives different forms of SL than plants do (Gutjahr, 2014; Boyer et al., 2014), and the *Rhizophagus irregularis* genome does not appear to contain orthologues of plant SL receptors (Tisserant et al., 2012).

On the other hand, SL is probably not the only molecule that AMF target for host-localization. Plants deficient in SL synthesis show a reduction in, but not the abolishment of, AMF colonization (Gómez-Roldán et al., 2008), while AMF non-host plants have also been shown secrete SL from their roots, albeit at lower levels (Goldwasser et al., 2008; Yoneyama et al., 2008). It seems plausible that SL-insensitive AMF can still colonize roots if encountered by chance, and SL merely functions to enhance host-guidance but is not essential for colonization. AMF species including *Gigaspora gigantean* and *Glomus mosseae*, and ectomycorrhizal fungal species including *Pisolithus tinctorius* and *Paxillus involutus* have been shown to prefer host roots over non-hosts or dead plants (Koske, 1982; Horan and Chilvers, 1990; Sbrana and Giovannetti, 2005). Since SL appears to be ubiquitously found in all plants, the presence of SL alone is not sufficient to dictate AMF colonization. Other root-derived AMF branching factors probably exist, but the situation is complicated since different compounds may have different effects on the same AMF, while the same compound may have different effects on different AMF (Nagahashi and Douds, 2000; Nagahashi and Douds, 2007). Different forms of SL may also have different attracting strengths and activities.

## Endoparasitic Nematodes

Another class of endoparasitic plant pathogens known to cause novel organ formation and developmental reprogramming of the host is the nematodes. The major nematode plant pathogens comprise the root-knot nematodes (RKNs, genus *Meloidogyne*), the cyst nematodes (CN, genera *Heterdera* and *Globodera*) and the pine-wilt nematodes (PWN, *Bursaphelenchus xylophilus*). Although RKNs and CNs appear to have evolved independently, both use infection mechanisms that have much in common. In both cases, second-instar juveniles (J2) roam freely in the soil searching for the roots of appropriate host plants. Once a suitable root has been identified, the J2s infect the root and inject effectors that reprogram the host’s vascular cells to form specialized feeding organs (Bartlem et al., 2014; Favery et al., 2016). RKNs stimulate host cells to undergo endoreduplication and form multi-nucleated giant cells, while CNs merge multiple host cells together to form syncytia (Siddique and Grundler, 2018). The nematodes then feed on these specialized organs and develop to maturity, whereupon females emerge from the roots to lay eggs and release the next generation to the environment.

J2 host-targeting behavior is therefore critical in plant parasitic nematode biology, and chemotaxis towards plant exudates has been associated with this behavior. Soybean, pea, potato, tomato, and rice root exudates have all been shown to attract J2s of various plant pathogenic nematodes (Papademetriou and Bone, 1983; Zhao et al., 2000; Reynolds et al., 2011; Xu et al., 2015; Yang et al., 2016; Čepulytė et al., 2018). Specifically, phenolics, flavonoids, glycoside, fatty acids, and diamines in exudates and volatiles from roots have been shown to act as nematode attractants (Chitwood, 2002; Zhao et al., 2007; Ohri and Pannu, 2010; Ali et al., 2011; Oota et al., 2019; **Table 2**). In addition, Arabidopsis seeds were also shown to attract RKN, suggesting RKN may interact with plant seeds as well aside from roots (Tsai et al., 2019). Furthermore, it was revealed that nematode attractants and repellents are produced not only by plants but also by nematodes themselves. Many plant-parasitic nematodes have been shown to produce ascarosides, a class of glycolipid-based signaling molecules synthesized almost exclusively by nematodes (Manosalva et al., 2015). Depending on the types and compositions, ascarosides can regulate the aggregation/dispersion of conspecifics or even other nematodes (Manohar et al., 2020). On the other hand, other compounds have also been documented to influence nematode behavior such as carbon dioxide; the amino acids arginine and lysine; phenolic acids; the plant hormones salicylic acid and gibberellic acid; the growth supplement ethephon; 6-dimethylallylaminopurine; and nitrate analogues (Pline and Dusenbery, 1987; Wang et al., 2009; Wang et al., 2010; Fleming et al., 2017; Hosoi et al., 2017; **Table 2**).

*Caenorhabditis elegans* has been established as a model organism for nematodes, and its genome, cell development pathway and nervous system have been extensively characterized. By examining elements conserved among *C. elegans* and plant pathogenic nematodes it may be possible to further expand our knowledge of pathogenic nematode behavior. Chemotaxis in nematodes is regulated by the amphid and phasmid sensory organs in their head and tail, respectively. In *C. elegans*, a pair of amphids acts as the main sensory organs, which contain twelve types of sensory neurons. By using laser ablation of individual or combinations of neurons, the corresponding stimulant signals being transmitted by each neuron can be identified (Mori, 1999; Rengarajan and Hallem, 2016). Despite the fact that the neural structures of plant-parasitic nematodes are at least somewhat conserved with *C.elegans*, molecular evidence suggests plant-parasitic nematodes likely evolved from fungivorous ancestors, which are likely evolutionarily distant from bacterivorous *C. elegans* (Quist et al., 2015). Cautions should be applied when inferring homology relationships between plant-parasitic nematodes and *C. elegans* to account for their evolutionary divergence and different foraging preferences.

Olfactory receptors are highly expressed in the sensory neurons and play important roles in sensing specific signals. For example, the AWA neuron expresses the ODR-10 receptor, which is responsible for diacetyl detection, and consequently *odr-10* mutants fail to detect diacetyl compounds (Sengupta et al., 1996). Currently, 194 putative olfactory receptor genes have been identified in the *C. elegans* genome (Taniguchi et al., 2014). Therefore, we performed homology searches to look for orthologues of *C. elegans* olfactory receptors in the genomes of plant pathogenic nematodes, including the RKNs *Meloidogyne incognita* and *Meloidogyne arenaria*, the CNs *Heterodera glycines* and *Globodera rostochiensis*, and the PWN *B*. *xylophilus* (**Table 3**). Interestingly, the majority of *C. elegans* olfactory receptors are not conserved among plant pathogenic nematodes, although the few receptor orthologues that are present may be informative in determining their chemotactic behaviors. The *B*. *xylophilus* genome contains orthologues of SRV-11 (pentanedione avoidance), SRV-12 (benzaldehyde attraction), SRSX-26 (butanone attraction), SRSX-32 (pyrazine attraction), SRSX-33 (pentanedione and pyrazine attraction), SRSX-37 (pentanedione attraction), SRT-18 and SRT-25 (diacethyl avoidance). Meanwhile, the *M*. *incognita* and *M*. *arenaria* genomes contain orthologues of SRG-37 (pyrazine attraction). It would be interesting to determine whether the functions of these receptors are conserved among pathogenic nematodes and similarly regulate chemotaxis. On the other hand, *H*. *glycines* and *G*. *rostochiensis* genomes contain no orthologues of *C. elegans* olfactory receptors. This surprisingly low level of conservation of olfactory receptors suggests plant pathogenic nematodes have independently evolved unique signalling pathways to detect chemical signals. Meanwhile, four *C. elegans* chemosensory gene orthologues were identified in *M. incognita* as *Mi-odr-1*, *Mi-odr-3*, *Mi-tax-2* and *Mi-tax-4*, where knockdown mutants showed defects in their attraction to root exudates, volatile compounds (alcohols, ketones, aromatic compounds, esters, thiazole, and pyrazine), non-volatile compounds (carbohydrates, phytohormones, organic acids, amino acids, and phenolics), as well as ascaroside signalling (Shivakumara et al., 2019; **Table 1**). These putative *M. incognita*-specific chemosensory signaling modules are likely to play important roles in host-targeting, and the identification of the corresponding olfactory receptors for these pathways may help identify specific RKN chemoattractants.

**Table 3:** **C***. elegans* olfactory receptor orthologues present in plant pathogenic nematodes, their predicted functions, and E-values of DNA sequence similarities.

C. elegans GPCR	Predicted function	Species	Orthologue	E-value
SRV-11	Pentanedione avoidance	*B. xylophilus*	BXY_0066100	3.00E-11
		*B. xylophilus*	BXY_1231200	4.00E-10
		*B. xylophilus*	BXY_0069300	2.00E-09
SRV-12	Benzaldehyde attraction	*B. xylophilus*	BXY_0066100	5.00E-10
		*B. xylophilus*	BXY_1231200	3.00E-07
		*B. xylophilus*	BXY_0069300	1.00E-06
SRSX-26	Butanone attraction	*B. xylophilus*	BXY_1070000	4.00E-10
		*B. xylophilus*	BXY_1013200	1.00E-07
SRSX-32	Pyrazine attraction	*B. xylophilus*	BXY_1070000	3.00E-10
		*B. xylophilus*	BXY_1013200	2.00E-07
		*B. xylophilus*	BXY_1013400	4.00E-05
		*B. xylophilus*	BXY_0557500	6.00E-04
		*B. xylophilus*	BXY_0027800	9.00E-04
SRSX-33	Pentanedione and pyrazine attraction	*B. xylophilus*	BXY_1070000	2.00E-06
		*B. xylophilus*	BXY_0809300	3.00E-06
		*B. xylophilus*	BXY_1013400	3.00E-05
		*B. xylophilus*	BXY_0557500	9.00E-05
		*B. xylophilus*	BXY_0027800	2.00E-04
SRSX-37	Pentanedione attraction	*B. xylophilus*	BXY_1013400	3.00E-09
SRT-18	Diacethyl avoidance	*B. xylophilus*	BXY_1024600	5.00E-20
SRT-25	Diacethyl avoidance	*B. xylophilus*	BXY_1024600	9.00E-20
SRG-37	Pyrazine attraction	*M. incognita*	Minc3s00775g17185	2.30E-02
		*M. arenaria*	tig00002579.g60974	5.90E-02

Homologies were determined using Protein BLAST from WormBase ParaSite (https://parasite.wormbase.org/index.html) with the protein sequences of the 194 C. elegans chemoreceptors described by Taniguchi et al. (2014) against the following proteome databases: Bursaphelenchus_xylophilus_prjea64437, Meloidogyne_arenaria_prjna438575 and Meloidogyne_incognita_prjeb8714, Globodera_rostochiensis_prjeb13504, and Heterodera_glycines_prjna381081 using the default setting.

## Outstanding Challenges and Future Perspectives

Aside from microorganisms, many arthropod species are also known to be endoparasitic and can manipulate their host plant’s developmental program during infection. Insects from the orders Hemiptera and Hymenoptera and mites from the superfamily Eriophyoidea include endoparasitic members that form galls. Similar to the ectopic organs formed by endoparasitic/endophytic microbes, galls induced by endoparasitic arthropods function as feeding organs and/or physical barriers for protection. Mechanisms that mediate arthropod-mediated galling through phytohormone manipulation have been characterized in great detail (Tooker and Helms, 2014; de Lillo et al., 2018). However, how arthropod parasites locate their host plants has been relatively poorly investigated, for various reasons. First, arthropods may not rely heavily on chemotaxis to find hosts. Endoparasitic arthropods typically have poor mobility and rely on random forces for locomotion, such as wind (Nault and Styer, 1969; Sabelis and Bruin, 1996). Other endoparasitic arthropods specialize in infecting a single long-lived host plant, where progenies can continue to infect the same host as their parents (Lindquist and Oldfield, 1996; Manson and Oldfield, 1996). Second, arthropod-induced galls are among the most structurally diverse, with 13,000 insect species documented to form plant galls. Galling behavior appears to have evolved in arthropods multiple times, possibly through horizontal gene transfer from symbiotic bacteria or fungi (Gullan et al., 2005; Raman et al., 2005). Therefore, no single model organism system may be sufficient to represent the molecular signalling mechanisms for chemotaxis in endoparasitic arthropods, and these behaviors may have to be addressed in a case-by-case fashion.

Another major challenge in the characterization of plant endoparasites and endophytes are tri-trophic and other interactions that involve more parties. In nature, it is likely that plants will simultaneously encounter several of the endoparasites and endophytes discussed above, considering the same chemicals may attract organisms from multiple taxons. The outcome of these complex interactions will not be easy to predict under controlled laboratory conditions. For example, *Fusarium solani*, a plant fungal pathogen related to *F. oxysporum*, has been shown to induce virulence genes in response to the isoflavanoid pisatin in host roots, which is made by plants during stress (Straney et al., 1994; Straney et al., 2002). Similarly, the plant pathogenic nematode *M. incognita* can be attracted to polyamines from plant root exudates, which are also known to be produced in stressed plants (Oota et al., 2019). It appears that pathogens from multiple taxa tend to favor stressed plants, making simultaneous infection or colonization very likely scenarios in nature. On the other hand, SL has been shown to not only promote hyphal branching in AMF but also the germination of parasitic plants of the genera *Striga* and *Orobanche* (Cook et al., 1966; Cardoso et al., 2011). The plant SL production levels fluctuate during the course of AMF infection, with SL-synthesis genes up-regulated during early infection, and down-regulated during later infection stages (López-Ráez et al., 2015; Kobae et al., 2018). Furthermore, host plants utilize overlapping signalling components in response to both AMF and rhizobia infections, suggesting the two processes may have evolved together (Hirsch and Kapulnik, 1998; Guinel and Geil, 2002; Vierheilig and Piché, 2002; Parniske, 2008). Plants inoculated with rhizobia also show reduced *Orobanche* infection (Mabrouk et al., 2007a; Mabrouk et al., 2007b; Mabrouk et al., 2007c), while SL-synthesis genes are up-regulated during rhizobia colonization (Breakspear et al., 2014; van Zeijl et al., 2015). These lines of evidence suggest the interactions between host plants, rhizobia, AMF, and parasitic plants mediated by SL require more elaborate analysis to decipher.

In general, it appears that most known attractants of plant endoparasites and endophytes consist of common compounds such as metabolites and plant hormones, instead of unique or unusual compounds. Currently it remains very difficult to use chemotactic behavior alone to explain endoparasites’/endophytes’ host range. The more likely explanation may be that soil microorganisms sense and respond to multiple chemoattractant simultaneously. Plants may also produce chemoattractants that are toxic to attracted microorganisms. Lauric acid has been shown to have different effects on *M. incognita* depending on concentrations (Dong et al., 2014). Abiotic environmental factors may also influence the behavior of soil microorganisms. Factors such as pH, ions and temperature, redox potential, chelating compounds, and electrical potential have been documented to affect the behavior of plant parasitic nematodes (Rasmann et al., 2012). Therefore, endoparasite/endophyte host-targeting behavior is likely to be complex, involving both biotic and abiotic factors. Nevertheless, the identification and characterization of chemoattractants can have practical applications in agriculture. These chemoattractants or repellants may be applied in fields directly to manipulate the microorganisms behaviors, and ultimately improve the growth of crop plants. The chemotactic behaviors of different organisms may even be combined, as *C. elegans* has been shown to be capable of carrying rhizobia bacteria to plant hosts through phoresis (Horiuchi et al., 2005). With the identification of more chemoattractants, more sophisticated agricultural application strategies may eventually be designed and implemented in the future.

## Data Availability Statement

The datasets presented in this study can be found in online repositories. The names of the repository/repositories and accession number(s) can be found in the article/supplementary material.

## Author Contributions

AY-LT and SS conceptualized the work. AY-LT, MO, and SS wrote the article.

## Funding

This work was supported by JSPS KAKENHI (Grant Numbers 16F16406, 16K14757, 17H03967, 18H05487, and 20H00422) awarded to SS.

## Conflict of Interest

The authors declare that the research was conducted in the absence of any commercial or financial relationships that could be construed as a potential conflict of interest.
